# Cytokeratin7 and cytokeratin19 expression in high grade cervical intraepithelial neoplasm and squamous cell carcinoma and their possible association in cervical carcinogenesis

**DOI:** 10.1186/s13000-017-0609-4

**Published:** 2017-02-17

**Authors:** Hojung Lee, Hyekyung Lee, Yong Kyun Cho

**Affiliations:** 1 Department of Pathology, Nowon Eulji Medical Center, Eulji University, 280-1 Hagye 1-dong, Nowon-gu, Seoul, 139-711 Korea; 2 Department of Pathology, Daejeon Eulji Medical Center, Eulji University, 95 Dunsanseo-ro, Seo-gu, Daejeon, 35233 Korea; 3 Department of Internal Medicine, Division of Infectious Diseases, Gachon University Gil Medical Center, 1198 Guwol-dong, Namdong-gu, Incheon, 405-760 Korea

**Keywords:** Cervical cancer, Squamocolumnar junction, HPV, CK7, CK19, p16

## Abstract

**Background:**

High risk human papillomavirus (HR HPV) infects cells at the squamocolumnar junction (SCJ) of the cervix, causing cancer. Cytokeratin (CK)7 is an SCJ marker, and stains cervical neoplasia. CK19 is a binding partner of CK7 and expressed in cervical cancer. Despite this possible association between CK7/CK19 and cervical cancer, not much is known about the mechanism of CK7/CK19 involvement in HR HPV-mediated cervical carcinogenesis.

**Methods:**

We analyzed the expression pattern of CK7, CK19, and p16 by using immunohistochemistry and HPV infection by in situ hybridization in 25 cases of high grade cervical intraepithelial neoplasia (CIN3) and in 30 cases of squamous cell carcinoma (SCC).

**Results:**

CK19, p16, and HPV expression was positive in all CIN3 and SCC cases. CK7 expression was positive in all CIN3 cases and in 20/30 (66%) SCCs. Each protein showed diffuse or patchy staining with topographic distinction. Patchy staining of CK7 and episomal HPV DNA overlapped in the upper layer of CIN3 and central portion of an invasive nest in the SCC, whereas patchy CK19 staining and integrated HPV DNA were usually noted in the lower layer of CIN3 and the periphery of the SCC nest. The p16 staining pattern coincided with that of CK19 in a subset of SCC.

**Conclusion:**

These results suggest that CK7 may be more related with viral episomal replication and CK19 with viral integration, contributing to viral replication and malignant transformation in HR HPV infected cells. In addition, coordinate CK7/CK19 staining may be used as a valuable marker for predicting physical status of HR HPV and E7 oncoprotein level in cervical tumor.

**Electronic supplementary material:**

The online version of this article (doi:10.1186/s13000-017-0609-4) contains supplementary material, which is available to authorized users.

## Background

More than 200 human papillomavirus (HPV) types have different epithelial tropisms and high risk (HR) HPV infects the cervical epithelium, causing cancer [1]. Discrete cell population at squamocolumnar junction (SCJ) of the cervix is hypothesized as the source of HR HPV infection and initiation site of cervical cancer [2–4]. SCJ cells are identified as residual embryonic cells vulnerable to neoplastic transformation [3]. Cytokeratin (CK)7, AGR2, CD63, MMP7, and GDA are known as SCJ cell markers [2, 5]. In normal-appearing SCJ cells positive for CK7, HPV E2 (an early marker of HPV infection) is expressed and HPV E6/E7 mRNA are detected, supporting the role of these cells in cervical carcinogenesis [4]. CK7 is also expressed in most cervical intraepithelial neoplasia (CIN) and carcinomas [2, 6]. Moreover, CK7 positive CIN1 is more likely to progress to CIN3 compared with CK7 negative CIN1, suggesting that CK7 is a predictive marker for CIN3 progress [6].

CK19 pairs with CK7 in simple epithelia and with CK8/18 in stratified squamous epithelial cells [7]. In the normal cervix, CK19 and CK7 stain endocervical columnar and reserve cells and CK19 additionally stains basal layer cells of ectocervix [8, 9]. Like CK7, CK19 is also expressed in CIN, squamous cell carcinoma (SCC), and adenocarcinoma [8, 9]. Thus, CK19 and CK7 positive endocervical reserve cells were suggested as progenitor cells of cervical cancer [8, 9] before the concept of SCJ cell emerged. Despite this common expression of CK7 and CK19 in the normal cervix and cervical neoplasia, little is known about the association between CK7 and CK19 and HPV in cervical carcinogenesis.

HPV DNA exists as episomal or integrated state in cervical neoplasia [10–13]. In CIN1, HR HPV genomes persist in episomal form, whereas in CIN3 and carcinomas, the majority of HPV genomes are found in integrated form [10, 12]. Integration of the HR HPV genome into the human chromosome disrupts viral E2 gene, a transcriptional repressor of the oncogenes E6 and E7, resulting in the stabilization of oncogene transcription [14]. E7 oncoprotein binds to retinoblastoma protein (Rb), leading to loss of cell-cycle control. Rb inhibits transcription of p16, a cyclin-dependent kinase inhibitor [15]. Functional inactivation of Rb by E7 results in p16 overexpression, and p16 is used as a surrogate marker for E7 protein [15]. Therefore, E2 and E7 expression is in the negative link in CIN [16].

During HPV life cycle, the viral replication and the expression of viral genes are closely tied to the differentiation program of host epithelial cell [1]. In this process, CK network appears to be reorganized and regulated by viral proteins [17]. Viral E4 protein is thought to participate in CK disruption, virus release and transmission [17]. Different viral protein expression according to the layer of CIN may be related with different CK expression patterns depending on the layer and the grade of CIN [9, 16]. Indeed the expression of E2 and E7, represented by p16 is mutually exclusive and shows topological distinction and association with specific CKs in CIN [16]. Comprehensive expression of CK subtypes had been described in CIN and cancers [9], but the association between HPV status and CK subtypes, especially CK7 and CK19 in cervical cancer has not been studied. Thus, in this study, we analyzed the expression of CK7, CK19, and p16, and physical status of HPV in CIN3 and SCC to investigate their possible association in cervical carcinogenesis.

## Methods

### Tissue samples

The list of CIN and SCC was obtained from archives in the Department of Pathology at Nowon Eulji Medical Center, Eulji University. We retrieved 25 CIN3 and 30 SCC cases collected by cone biopsy, loop electrosurgical excision procedure (LEEP), or hysterectomy. The age of all CIN3 and SCC patients ranged from 27 to 79 years and the mean was 48 years. The stage of 30 SCC patients was classified into 11 cases of stage Ia, 10 stage Ib, 5 stage II, 3 stage III, and 1 stage IV according to the International Federation of Gynecology and Obstetrics staging. Ten non-neoplastic cervical specimens from the uteri removed for non-cervical pathologic conditions were also included. After reviewing all hematoxylin and eosin (HE) stained slides from each case, representative blocks for each case were selected. In two cases of CIN3 (LEEP) and one case of SCC (LEEP), immunohistochemical evaluation was not available in the slides made from initially selected blocks by repeat cutting due to technical reasons and other blocks for the same case were used for immunohistochemistry. This study was performed with the approval of the Institutional Review Board of Nowon Eulji Medical Center.

### Immunohistochemistry

Immunohistochemical staining was performed using Dako Autostainer (DakoCytomation, Carpinteria, CA, USA). Four micron tissue sections were cut from selected blocks and positioned on poly-L-lysine coated slides. After deparaffinization and rehydration, antigen retrieval was performed using citrate buffer (pH 6.0) at 121 °C for 10 min. Endogenous peroxidase activity was blocked by 3% hydrogen peroxide for 5 min. The primary antibodies used in this study were CK7 (Dako, Carpinteria, CA, 1:100), CK19 (Dako, 1:100), and p16 (Dako p16INK4a kit). Color was developed with diaminobenzidine, and the slides were counterstained with hematoxylin.

### Immunostaining assessment

CK7 and CK19 staining was interpreted as positive when approximately five to six contiguous cells showed cytoplasmic and/or membranous block staining [6]. With p16, nuclear or nuclear and cytoplasmic block staining was considered positive [18]. The immunoreactivity of CK7, CK19, and p16 was determined by assessing the staining intensity and percentage of stained cells. The staining intensity was rated as weak, moderate, and strong. The percentage of positive cells was scored as follows: negative – staining in less than 1% of cells, patchy – staining in 1% to 40% of cells, and diffuse – staining over more than 40% of cells.

### In situ hybridization

In situ hybridization (ISH) was performed using the INFORM HPV III Family 16 Probe (B) (Ventana Medical Systems, Tucson, AZ, USA), according to the manufacturer’s recommendations. The probe cocktail has demonstrated affinity for the following HR HPV genotypes: 16, 18, 31, 33, 35, 39, 45, 51, 52, 56, 58, and 66. The HPV signal patterns were classified as the episomal or integrated form, found within the nuclei of cervical epithelial cells. The episomal HPV was defined as large globular dense nuclear staining. The integrated HPV was defined as discrete dot-like signals or scattered tiny particles in the nuclei.

## Results

### Expression of CK7 and CK19 in non-neoplastic cervix

**Fig. 1:**
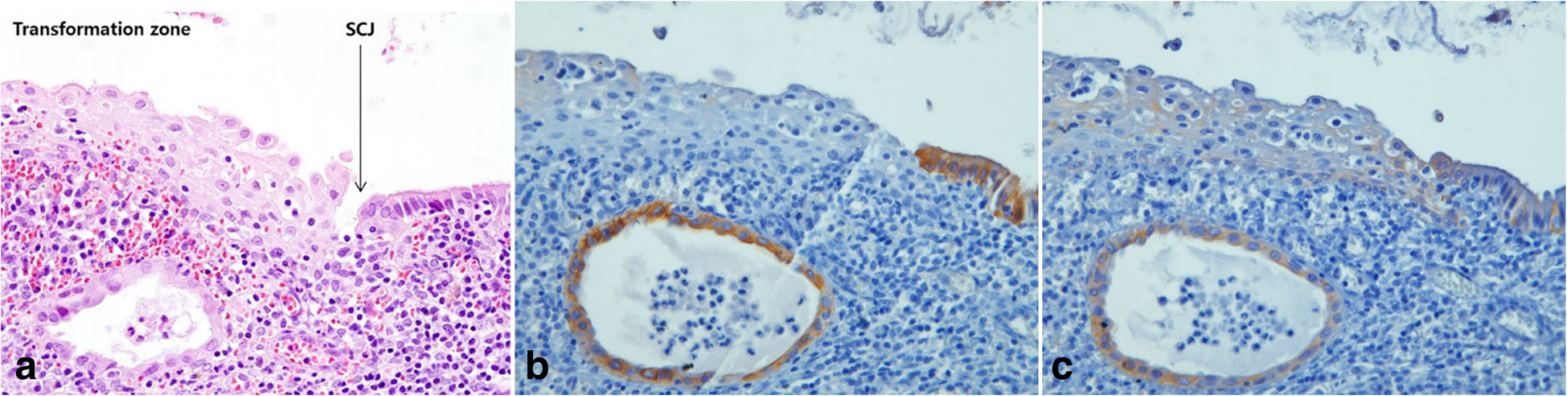
Expression of CK7 and CK19 in non-neoplastic cervix. HE staining shows the transformation zone and SCJ cells (*arrow*) (**a**). Strong CK7 staining is seen in the columnar cells at the SCJ with faint staining of CK7 in the squamous cells at the transformation zone (**b**). Weak staining of CK19 is seen in the columnar cells at the SCJ and the squamous cells at the transformation zone (**c**). Original magnifications: x400

In the non-neoplastic cervix (Fig. 1a), CK7 staining was strong in the columnar cells at the SCJ, faintly seen in the cells at the transformation zone (Fig. 1b), and negative in squamous cells at the ectocervix. CK19 was weakly stained in the columnar cells at the SCJ, squamous cells at the transformation zone (Fig. 1c), and basal layer cells at the ectocervix.

### Expression of CK7, CK19, p16, and HPV in CIN3

**Fig. 2:**
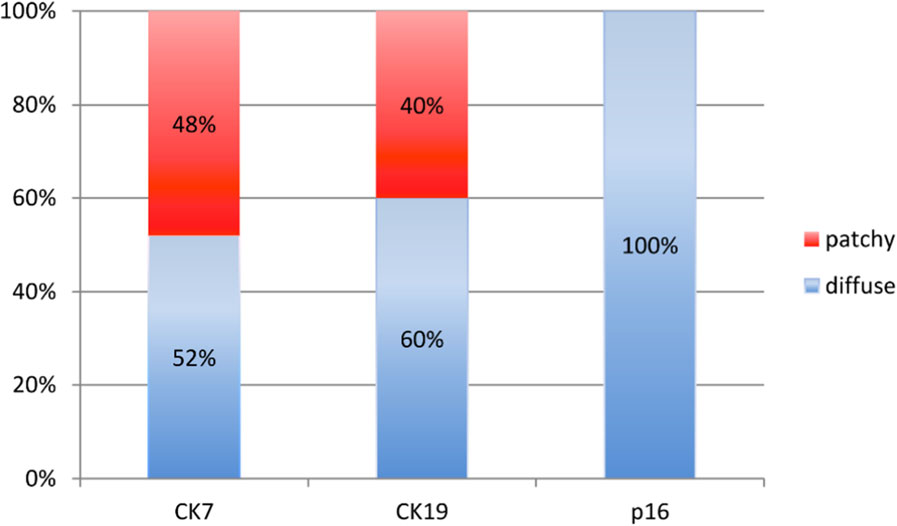
The result of CK7, CK19, and p16 expression in CIN3

**Fig. 3:**
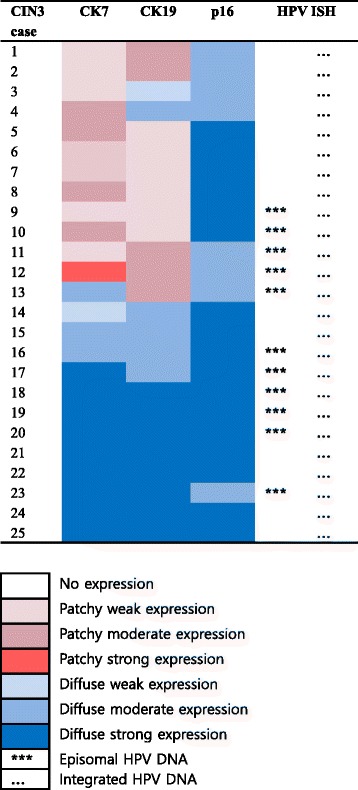
Expression profile of CK7, CK19, p16, and HR HPV in CIN3

**Fig. 4:**
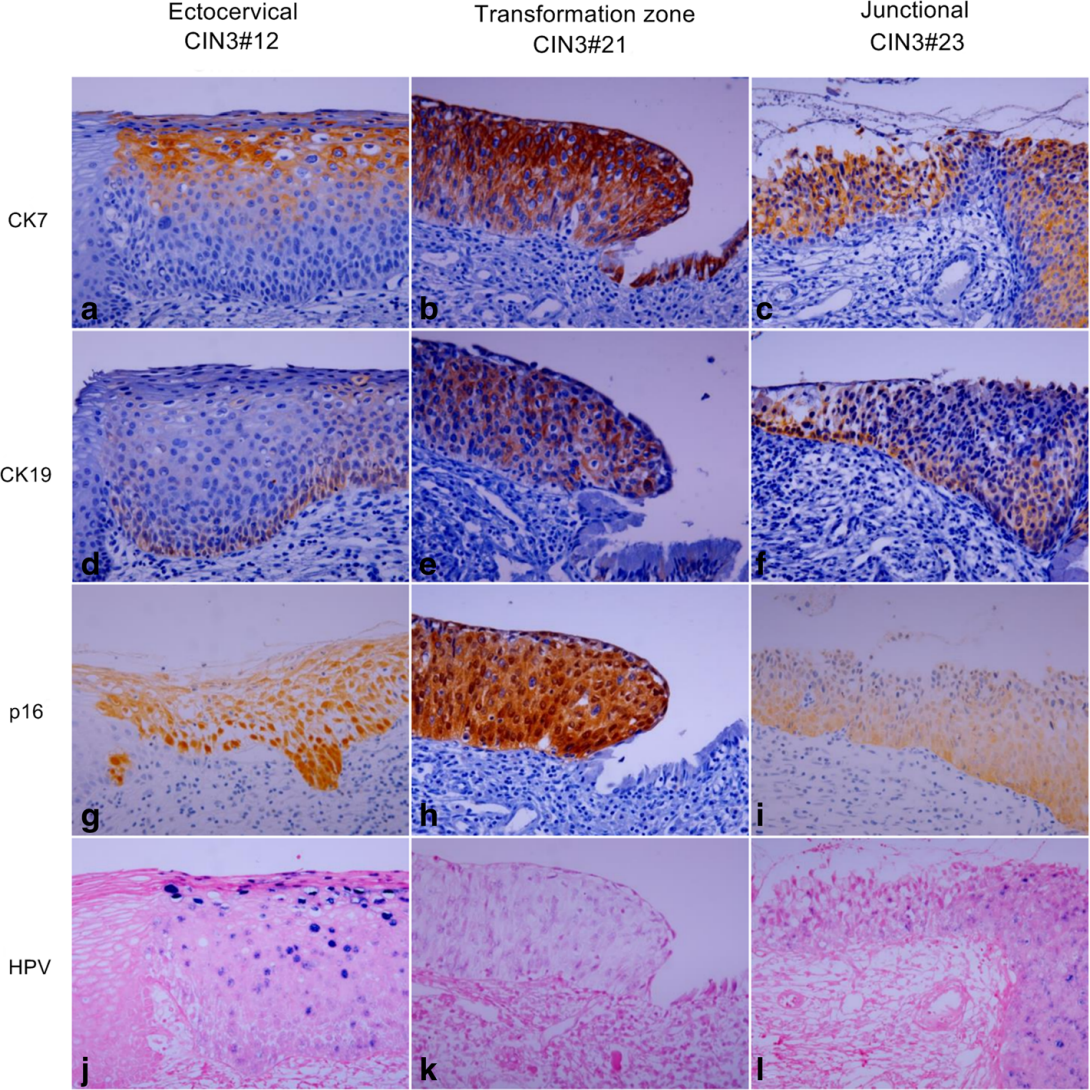
Expression pattern of CK7, CK19, p16, and HR HPV in CIN3. HE staining shows representative CIN3s developing in the ectocervix (CIN3#12) (**a**), transformation zone (CIN3#21) (**b**), and SCJ (CIN3#23) (**c**), respectively. CIN3#12 shows patchy staining of CK7 in the upper layer (**d**), patchy staining of CK19 in the lower layer (**g**), diffuse staining of p16 (**j**), mixture of episomal and integrated HPV with remarkable episomal form in the upper layer (**m**). CIN3#21 and CIN3#23 show diffuse staining of CK7 (**e** and **f**), CK19 (**h** and **i**), and p16 (**k** and **l**). HPV is present in integrated form in CIN3#21 (**n**) and mixed episomal and integrated form in CIN3#23 (**o**). Original magnifications: x400

The results of immunohistochemistry for CK7, CK19, and p16 in CIN3 are shown in Fig. 2 and their detailed expression patterns and HPV status are demonstrated in Fig. 3. The expression of CK7, CK19, and p16 was positive in all of 25 CIN3 cases. There were 12 (48%) patchy stained cases of CK7, and 10 (40%) of CK19, topographically distinct. Patchy CK7 expression was usually seen in the upper layer of CIN3, most intensely in the superficial layer and gradually weaker in the lower layer. In some cases of CIN3 located in the ectocervix (CIN3#12, Fig. 4a), CK7 staining was strong in the cells in the upper layer (Fig. 4d). Conversely, patchy CK19 expression was most intense in the basal layer of CIN3 and gradually weaker toward the upper layer (Fig. 4g). Diffuse staining of CK7 was seen in 13 (52%) and 15 (60%) cases of CK19, with homogeneous expression in entire layer of CIN3 located at the transformation zone (CIN3#21, Fig. 4b, e and h) and the SCJ (CIN3#23, Fig. 4c, f and i). p16 expression was diffuse in all CIN3 cases with moderate (Fig. 4j and l) to strong intensity (Fig. 4k).

HPV was detected in all CIN3 cases including 11 (44%) cases with combined episomal and integrated form and 14 (56%) cases with integrated form. Episomal HPV DNA was usually seen in the upper layer corresponding with CK7 staining area (Fig. 4m and o), while integrated HPV DNA was noted in entire layer (Fig. 4n).

### Expression of CK7, CK19, p16, and HPV in SCC

**Fig. 5:**
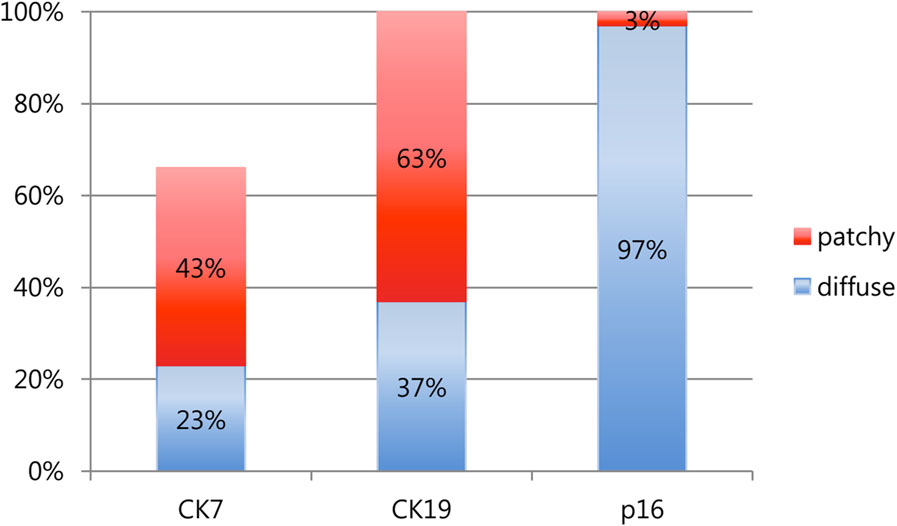
The result of CK7, CK19, and p16 expression in squamous cell carcinoma

**Fig. 6:**
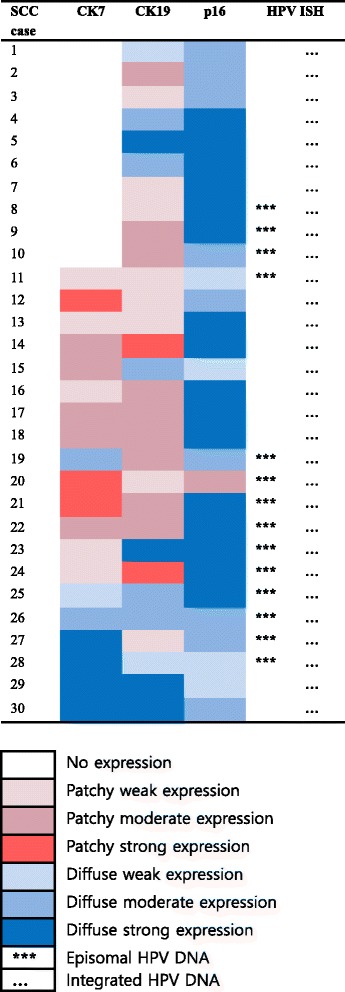
Expression profile of CK7, CK19, p16, and HR HPV in squamous cell carcinoma

**Fig. 7:**
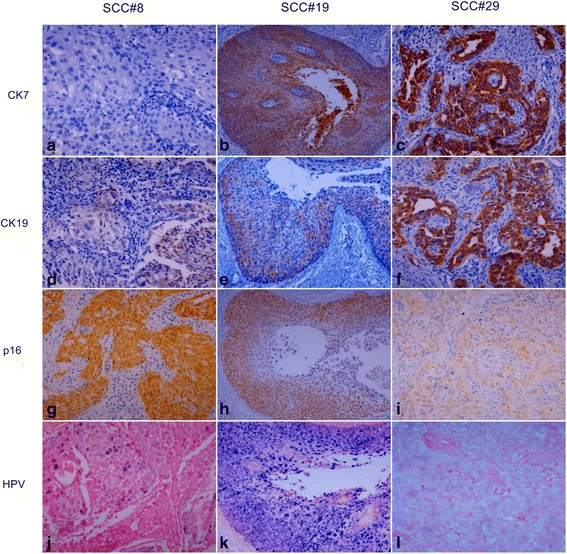
Expression pattern of CK7, CK19, p16, and HR HPV in squamous cell carcinoma (SCC). HE staining shows SCC with solid tumor nests (SCC#8) (**a**), SCC with cystic change (SCC#19) (**b**), and SCC with glandular differentiation (SCC#29) (**c**). CK7 staining is negative in SCC#8 (**d**) and diffusely positive in SCC#19 (**e**) and SCC#29 (**f**). CK19 staining pattern is patchy in SCC#8 (**g**) and SCC#19 (**h**), and diffuse in SCC#29 (**i**). p16 staining is diffusely positive with variable intensity in SCC#8 (**j**), SCC#19 (**k**), and SCC#29 (**l**). HPV is present in mixture of episomal and integrated form in SCC#8 (**m**) and predominant episomal form in the cystic tumor nest and cellular debris shedding within the cystic space of SCC#19 (**n**), and integrated form in SCC#29 (**o**). Original magnifications: (**a**), (**c**), (**d**), (**f**), (**g**), (**i**), (**j**), (**l**), (**m**), and (**o**) x400; (**b**), (**e**), (**h**), (**k**) and (**n**) x200

The results of immunohistochemistry for CK7, CK19, and p16 in SCC are shown in Fig. 5 and their detailed expression patterns and HPV status are demonstrated in Fig. 6. While the expression of CK19 and p16 was positive in all 30 SCC cases, CK7 expression was positive in 20 cases (67%) and negative in 10 cases (33%). Patchy staining of CK7 was seen in 13 (43%) and 19 cases (63%) of CK19. Of the typical SCCs (13 cases, 43%) showing solid tumor nests with/without keratinous pearl (SCC#8, Fig. 7a), CK7 staining was positve in 8 cases (62%) and negative (Fig. 7d) in 5 cases while CK19 staining was positive (Fig. 7g) in all cases. Interestingly, in the case of SCC#28, CK7 staining was noted in a keratinizing tumor nest. Topologically distinctive staining pattern of CK7 and CK19 was seen in SCC with central cystic space within the tumor nest, such as SCC#19. In SCC#19 (Fig. 7b), CK7 staining was diffusely positive in overall tumor nest but stronger in the cells of inner portion of the nest and cellular debris shedding within the space (Fig. 7e). However, CK19 positive cells were patchily present along the periphery of the invasive tumor nests (Fig. 7h) ﻿(Addittional File 1: Figure S1). The central cystic space within the tumor nest was seen in 14 cases (47%) and 10 of 14 cases (71%) were positive for CK7. In SCC showing infiltrating cord-like or branching growth pattern (Fig. 7c), CK7 and CK19 staining was both diffuse and strong in the tumor cells, and their staining was intensified in the invasive front and tumor cells with glandular differentiation (SCC#29, Fig. 7f and i). This growth pattern was seen in 3 cases (10%) and 2 of 3 cases (67%) were positive for CK7. Despite strong CK7 and CK19 expression in a subset of SCC, overall staining of CK7 and CK19 in SCC was weaker than that in CIN3

The expression of p16 was also weaker in SCC compared with CIN3, with even one patchy stained case (SCC#20, Fig. 6). In SCC#19, the staining of p16 was diffuse but disappeared toward the inner portion of the nest (Fig. 7k). It was notable that in SCC with diffuse and strong CK7 staining, p16 staining was weak (SCC#29, Fig. 7l), whereas in SCC with no CK7 staining, p16 staining was strong (SCC#8, Fig. 7j), suggesting an inverse correlation between CK7 and p16 in SCC, although not consistent in all cases.

HPV was positive in all SCC samples, displaying mixed episomal and integrated form in 14 (47%) and integrated form in 16 (53%) of 30 SCC cases. The cases with integrated HPV DNA revealed no topographic distinction. In SCC#19, an episomal form was seen in the entire tumor nest but more frequently in central portion of the nest, overlapping with the expression of CK7 (Fig. 7n). However, an episomal HPV was not always concordant with CK7 expression because HPV with the integrated form only was also seen in tumor cells with strong CK7 positivity such as SCC#29 (Fig. 7o). Inversely, in CK7 negative case such as SCC#8, an episomal form of HPV DNA was also noted (Fig. 7m).

## Discussion

In this study, we found consistent expression of CK19, p16, and HR HPV in CIN3 and SCC cases. However, CK7 expression was lost in one-third of SCC cases, although its expression was positive in all CIN3 cases. CK7 and CK19 showed topographic distinction in patchy stained CIN3 and SCC cases. In addition, CK7 and CK19 staining appeared to be more linked with episomal and integrated forms of HPV, respectively. The expression of CK7 or CK19 has been intensely studied in HPV mediated oropharyngeal SCC as well as cervical neoplasia [2–4, 19, 20]. However, the mechanism of CK7 and CK19 involvement in HPV positive cancer is largely unknown. Only a few studies provide clues to this by examining the relationship between HPV16 E7 and CK19/CK7 in cervical cancer cell lines [21–23].

In SiHa cells, HPV16 E7 mRNA interacts with the 6-mer peptide SEQIKA of CK7 and E7 mRNA translation is inhibited [22]. In contrast, CK19 promotes the translation of E7 mRNA into the oncoprotein by binding to the CK7 SEQIKA peptide [23]. Favia et al [21] show that in SiHa cells, which have CK19 and CK7, higher E7 protein level is detected notwithstanding low copies of HPV16 DNA per cell compared to CaSki cells, which have only CK7 and shows low E7 protein in spite of a high number of HPV16 DNA per cell [21]. These results suggest a differential role of CK7 and CK19 on HPV16 E7 oncoprotein expression, such as CK7 as a protector of E7 transcript and CK19 as an enhancer of E7 mRNA translation into the oncogenic product [21]. The present study showed mutually exclusive expression of CK7 and CK19 and concordant expression of CK19 and p16 in a subset of CIN3 and SCC, in agreement with the literature.

In CIN1, CK7 staining is predominantly strong in the surface epithelial cells [3, 6]. As the lesion is proceeding to CIN2/3, CK7 stain is extending down toward the basal layer (top down expression), recapitulating cervical development during fetal period [3, 9]. Given that CK7 is consistently stained in the SCJ cells and the upper layer cells in immature metaplasia and all grades of CIN [2–4, 16], it would be assumed that CK7 positive cells in the luminal side of an invasive cancer nest (SCC#19) might be the cells line in this sequence. However, why these CK7 positive cells persist in the upper layer of cervical lesions and how they participate in the neoplastic process are unclear. Presumably, HR HPV infected SCJ cells induce proliferation of CK7 positive cells more basally, functioning as progenitor cells of cervical neoplasia [3]. Otherwise, prior to or in concert with HR HPV infection, residual embryonic SCJ cells may migrate towards adjacent squamous cells upon injury or inflammation, followed by downward clonal expansion of CK7 positive metaplastic or dysplastic cells, similar to that seen in Barrett’s esophagus [24].

CK19 expression extends upward from the basal to superficial layer (bottom up expression) as CIN grade is increasing [8, 9]. p16 stains similarly to CK19 with the severity of CIN [12]. This might be related to the function of CK19 promoting the translation of E7 mRNA into the oncoprotein as previously discussed [21, 23]. This upward expression pattern of CK19 supports the traditional view that HR HPV first infects exposed basal cells and transformed basal cells divide and migrate upward following squamous epithelial differentiation program [1, 25]. Indeed CK19 is a stem cell marker in skin and squamous epithelia-lined mucosa such as the oral cavity [26, 27]. CK19 staining is negatively associated with the expression of involucrin, terminal differentiation protein in oral epithelium, suggesting that CK19 expression may be linked to the retention of stem cell character [26]. The stem cells retain a high capacity for self-renewal and a large proliferative potential [27]. Therefore, the basal cells of the ectocervix would be preferential site for HPV infection if viruses can access these cells probably through a microwound [1]. Presumably, basal expression of CK19 and HPV in CIN3#12 of the present study reflects basal cells infected by HR HPV in the ectocervix, similar to skin and oral cavity.

In the present study, CK19 and CK7 were both stained at the cells of the transformation zone and SCJ of the non-neoplastic cervix, although staining intensity of CK19 was weaker than that of CK7 in SCJ. In normal human organs, co-expression of CK7 and CK19 occurs in hepatic progenitor cells, which are capable of differentiating into hepatocytes and cholangiocytes [28]. If SCJ cells of the cervix function as progenitor cells, like hepatic progenitor cells, SCJ cells would be capable of differentiating into squamous cell and columnar cell and evolving into CIN showing squamous and columnar cell feature, such as CIN3#21 and CIN3#23, respectively. We found that CK7 and CK19 were diffusely co-expressed in CIN3#21 and CIN3#23, which occurred in the transformation zone and SCJ, respectively. CK7 and CK19 expression was mutually exclusive and patchy in CIN3#12, which occurred in the ectocervix close to the transformation zone. These results suggest that the expression pattern of CK7 and CK19 might indicate the location of the cervix in which CIN3 arises, although not consistently. In SCC, the expression pattern of CK7 and CK19 was variable depending on the tumor nests within the same tumor; thus the initial cancer site could not be predicted. Nevertheless, SCC showing glandular differentiation and diffuse expression of CK7 and CK19 such as SCC#29 might arise from SCJ. Likewise, SCC showing patchy expression for CK19 with/without CK7 might reflect the cancer developed from ectocervix, where CK19 stain is consistently present in basal layer cells, while CK7 stain is sporadically noted in the upper layer close to the SCJ. Thus, presumably CK19 is a common progenitor cell marker of cervix cancer developing in the SCJ and ectocervix.

In cervical cancer, HR HPV is mainly detected in integrated form [10, 11, 29], although there are studies showing dominant episomal form or mixed episomal and integrated form in cancer biopsy specimens [12, 13]. HPV integration seems to contribute to the stabilization of oncogene transcription [29]. Thus, diffuse expression of p16 in CIN3 and SCC in the present study might represent stable E7 production through HPV integration. In the present study, integration of HR HPV was found in all cases and episomal HPV DNA was observed in 44% (11/25 cases) of CIN3 and 47% (14/30 cases) of SCC. Both episomal and integrated HPV DNA coexist in the same tumor cell nuclei [11]. E2 is not only a transcriptional repressor of E6 and E7, but also required for viral genome replication [1, 16]. Therefore, E2 protein from coexisting intact episomal E2 gene could replace the regulatory function of an integrated defective E2 gene [11]. In some cases of the present study, episomal HPV overlapped with CK7 expression in the upper layer of CIN3 (CIN3#12) and SCC with cystic tumor nest (SCC#19), suggesting possible association between CK7 and viral genome replication. Although CK7 is known to be involved in regulating E7 transcript [21], whether CK7 plays a role in the process of viral replication is unclear. However, considering that CKs including CK7 regulate protein biosynthesis through localization of glucose transporter and activation of mTOR pathway [30], CK7 might be essential for production of proteins for viral replication and assembly. Moreover, CK7 is commonly expressed in most secretory cells of human organ [31], thus CK7 positive cells with secretory property in the upper layer of CIN3 and inner surface of SCC nests would be advantageous for viral release after viral particles are generated.

There are still questions left unresolved in this study. The first is how the cells with stem cell character were simultaneously present in the upper layer and the lower layer of CIN3 showing CK7 and CK19 positivity, respectively as shown in CIN3#12. One possible explanation would be concurrent neoplastic transformation of CK7 positive cells in the upper layer derived from SCJ and CK19 positive basal stem cells in the lower layer infected by different or multiple HR HPV [32, 33]. CIN3#12 in this study might imply coexistent CIN lesion from different cell clones lying in the upper and lower layer cells infected by different HPV types [33], although HPV typing is required.

The second question is why CK7 expression was not observed in 10 of 30 cases of SCC whereas CK19 expression was retained even with a patchy pattern in all SCC cases. This might be related to a basically different expression level of CK7 and CK19 depending on the location in the cervix in which SCC arises. As CK7 was not detected in the ectocervix distant to SCJ, SCC developed from this area would show only CK19 positivity. However, it remains to be explored why CK7 and CK19 did not co-localize to the same tumor cell populations despite their pairing property.

The last one is why the expression of CK19 as well as CK7 was lower or weaker in SCC compared to CIN3. Changes of CK7 and CK19 expression level in invasive cancer compared with CIN3 might be related to migration capacity of cancer cells [34] or asymmetric division of CK7 and/or CK19 positive progenitor cells during cancer progression [35].

## Conclusions

In conclusion, our results support earlier studies in which CK7 might be a predictive marker for HPV infection and CIN3 progress. In addition, consistent expression of CK19 and p16, accompanied by HPV integration in CIN3 and SCC tissues supports the idea that CK19 may promote E7 oncoprotein production, contributing carcinogenic events. We suggest that coordinate CK7/CK19 staining may be used as a valuable marker for predicting the physical status of HR HPV and E7 level in cervical tumor.

## Additional file

Additional file 1:
**Figure S1.** Low power view (x40) of HE staining (a) and CK7 (b) CK19 (c) p16 (d) and HR HPV (e) expression pattern of SCC#19. (PPTX 3814 kb)

## Abbreviations

CIN: Cervical intraepithelial neoplasia
CK: Cytokeratin
HR HPV: High risk human papillomavirus
ISH: In situ hybridization
LEEP: Loop electrosurgical excision procedure
Rb: Retinoblastoma protein
SCC: Squamous cell carcinoma
SCJ: Squamocolumnar junction
