# Erratum to: Cytokeratin7 and cytokeratin19 expression in high grade cervical intraepithelial neoplasm and squamous cell carcinoma and their possible association in cervical carcinogenesis

**DOI:** 10.1186/s13000-017-0632-5

**Published:** 2017-05-22

**Authors:** Hojung Lee, Hyekyung Lee, Yong Kyun Cho

**Affiliations:** 1 Department of Pathology, Nowon Eulji Medical Center, Eulji University, 280-1 Hagye 1-dong, Nowon-gu, Seoul 139-711 Korea; 2 Department of Pathology, Daejeon Eulji Medical Center, Eulji University, 95 Dunsanseo-ro, Seo-gu, Daejeon, 35233 Korea; 3 Department of Internal Medicine, Division of Infectious Diseases, Gachon University Gil Medical Center, 1198 Guwol-dong, Namdong-gu, Incheon, 405-760 Korea

## Erratum

The original version of this article [1] unfortunately contained incorrect figure files. Figures 4 and 7 in main body which have been replaced with new version of these figures. The corrected Figures include 3 added HE (a,b,c) and total 15 (a-o) figures, each.

**Fig. 4:**
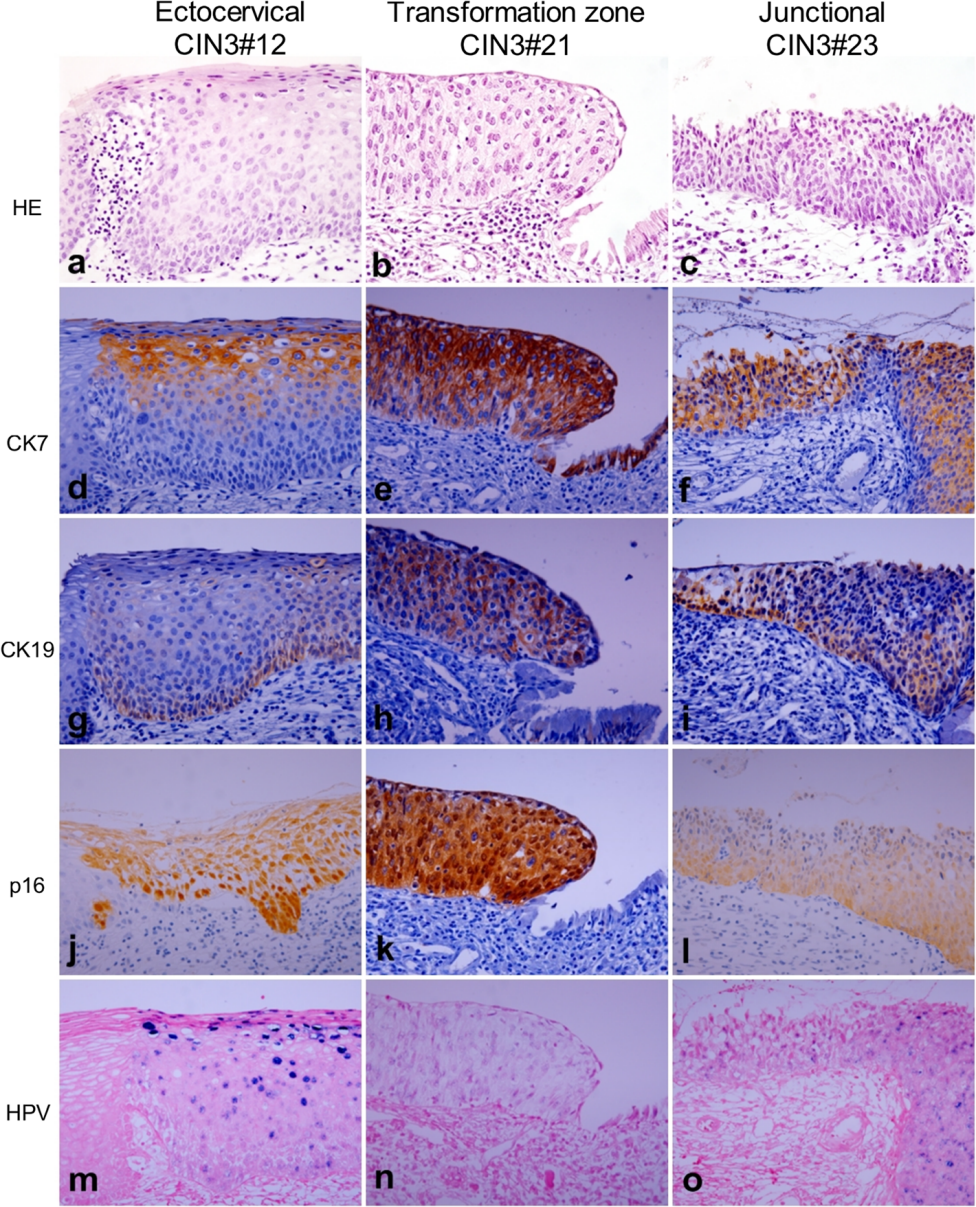
Expression pattern of CK7, CK19, p16, and HR HPV in CIN3. HE staining shows representative CIN3s developing in the ectocervix (CIN3#12) (**a**), transformation zone (CIN3#21) (**b**), and SCJ (CIN3#23) (**c**), respectively. CIN3#12 shows patchy staining of CK7 in the upper layer (**d**), patchy staining of CK19 in the lower layer (**g**), diffuse staining of p16 (**j**), mixture of episomal and integrated HPV with remarkable episomal form in the upper layer (**m**). CIN3#21 and CIN3#23 show diffuse staining of CK7 (**e** and **f**), CK19 (**h** and **i**), and p16 (**k** and **l**). HPV is present in integrated form in CIN3#21 (**n**) and mixed episomal and integrated form in CIN3#23 (**o**). Original magnifications: x400

**Fig. 7:**
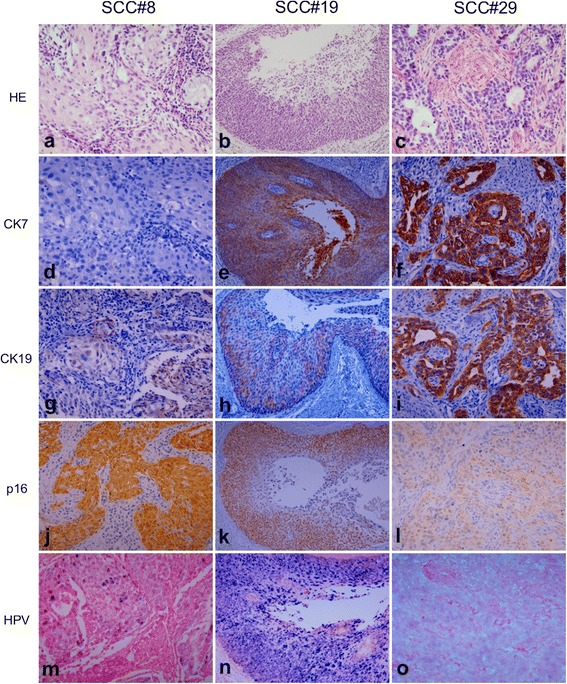
Expression pattern of CK7, CK19, p16, and HR HPV in squamous cell carcinoma (SCC). HE staining shows SCC with solid tumor nests (SCC#8) (**a**), SCC with cystic change (SCC#19) (**b**), and SCC with glandular differentiation (SCC#29) (**c**). CK7 staining is negative in SCC#8 (**d**) and diffusely positive in SCC#19 (**e**) and SCC#29 (**f**). CK19 staining pattern is patchy in SCC#8 (**g**) and SCC#19 (**h**), and diffuse in SCC#29 (**i**). p16 staining is diffusely positive with variable intensity in SCC#8 (**j**), SCC#19 (**k**), and SCC#29 (**l**). HPV is present in mixture of episomal and integrated form in SCC#8 (**m**) and predominant episomal form in the cystic tumor nest and cellular debris shedding within the cystic space of SCC#19 (**n**), and integrated form in SCC#29 (**o**). Original magnifications: (**a**), (**c**), (**d**), (**f**), (**g**), (**i**), (**j**), (**l**), (**m**), and (**o**) x400; (**b**), (**e**), (**h**), (**k**) and (**n**) x200

The new version of these files are included below.

Furthermore, Additional File 1 has been updated to remove Fig. 3 and 6 which have already been included in the main body of the article. The original version of this article has been updated to reflect the above changes. 

## Additional file

Additional file 1: Figure S1. Low power view (x40) of HE staining (a) and CK7 (b) CK19 (c) p16 (d) and HR HPV (e) expression pattern of SCC#19 (PPTX 3720 kb)
