# Supplementary material for: Cytokeratin7 and cytokeratin19 expression in high grade cervical intraepithelial neoplasm and squamous cell carcinoma and their possible association in cervical carcinogenesis
Source: Diagn Pathol. 2017 Feb 17;12:18. doi: 10.1186/s13000-017-0609-4 (PMC5316189; doi:10.1186/s13000-017-0609-4)
Supplement: Additional file 1: — Figure S1. Low power view (x40) of HE staining (a) and CK7 (b) CK19 (c) p16 (d) and HR HPV (e) expression pattern of SCC#19. (PPTX 3814 kb) [file 13000_2017_609_MOESM1_ESM.pptx]

## Slide 1
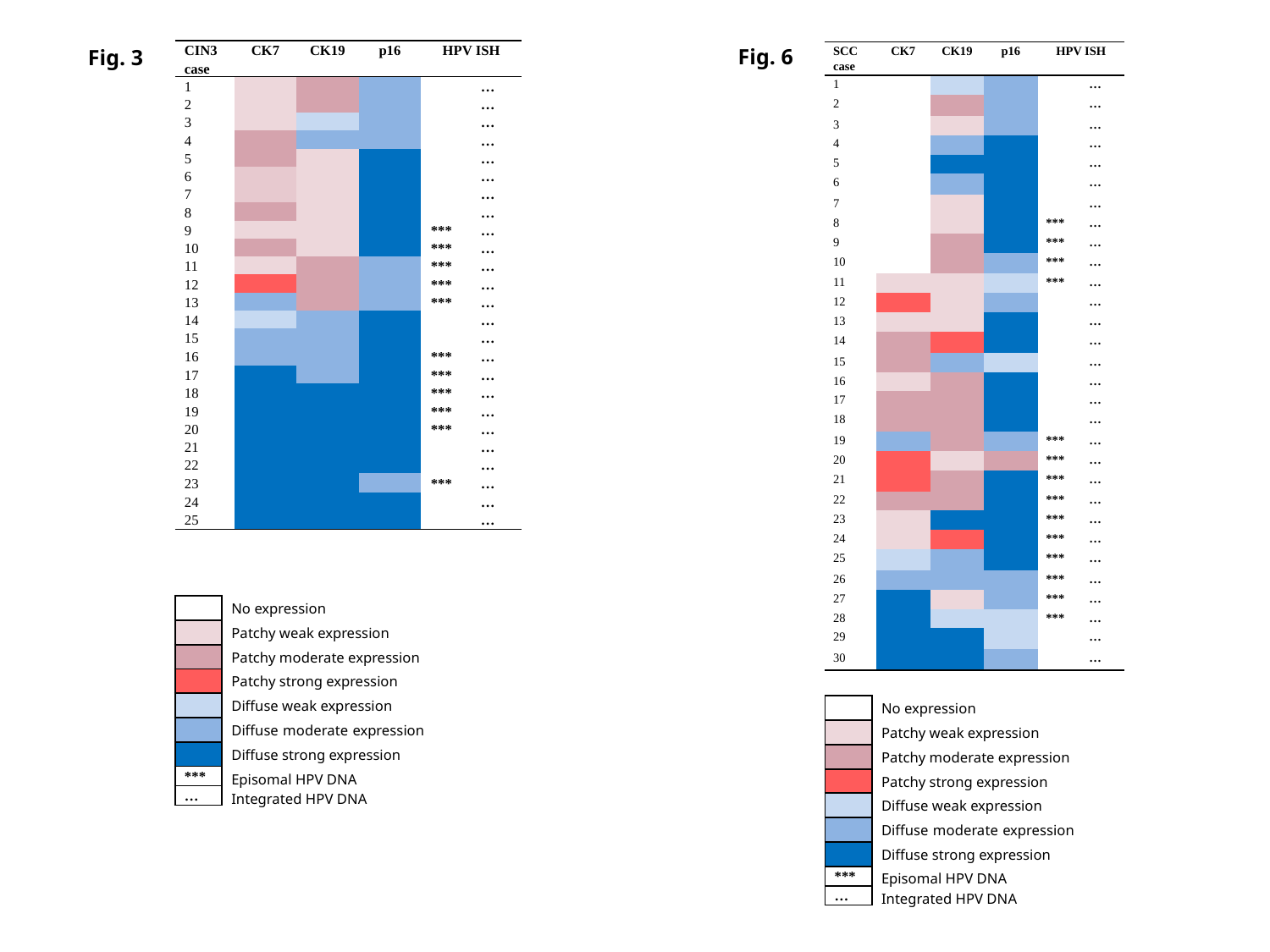

Fig. 6
Fig. 3
| CIN3 case | CK7 | CK19 | p16 | HPV ISH | |
| --- | --- | --- | --- | --- | --- |
| 1 | | | | | … |
| 2 | | | | | … |
| 3 | | | | | … |
| 4 | | | | | … |
| 5 | | | | | … |
| 6 | | | | | … |
| 7 | | | | | … |
| 8 | | | | | … |
| 9 | | | | \*\*\* | … |
| 10 | | | | \*\*\* | … |
| 11 | | | | \*\*\* | … |
| 12 | | | | \*\*\* | … |
| 13 | | | | \*\*\* | … |
| 14 | | | | | … |
| 15 | | | | | … |
| 16 | | | | \*\*\* | … |
| 17 | | | | \*\*\* | … |
| 18 | | | | \*\*\* | … |
| 19 | | | | \*\*\* | … |
| 20 | | | | \*\*\* | … |
| 21 | | | | | … |
| 22 | | | | | … |
| 23 | | | | \*\*\* | … |
| 24 | | | | | … |
| 25 | | | | | … |
| SCC case | CK7 | CK19 | p16 | HPV ISH | |
| --- | --- | --- | --- | --- | --- |
| 1 | | | | | … |
| 2 | | | | | … |
| 3 | | | | | … |
| 4 | | | | | … |
| 5 | | | | | … |
| 6 | | | | | … |
| 7 | | | | | … |
| 8 | | | | \*\*\* | … |
| 9 | | | | \*\*\* | … |
| 10 | | | | \*\*\* | … |
| 11 | | | | \*\*\* | … |
| 12 | | | | | … |
| 13 | | | | | … |
| 14 | | | | | … |
| 15 | | | | | … |
| 16 | | | | | … |
| 17 | | | | | … |
| 18 | | | | | … |
| 19 | | | | \*\*\* | … |
| 20 | | | | \*\*\* | … |
| 21 | | | | \*\*\* | … |
| 22 | | | | \*\*\* | … |
| 23 | | | | \*\*\* | … |
| 24 | | | | \*\*\* | … |
| 25 | | | | \*\*\* | … |
| 26 | | | | \*\*\* | … |
| 27 | | | | \*\*\* | … |
| 28 | | | | \*\*\* | … |
| 29 | | | | | … |
| 30 | | | | | … |
| | No expression |
| --- | --- |
| | Patchy weak expression |
| | Patchy moderate expression |
| | Patchy strong expression |
| | Diffuse weak expression |
| | Diffuse moderate expression |
| | Diffuse strong expression |
| \*\*\* | Episomal HPV DNA |
| … | Integrated HPV DNA |
| | No expression |
| --- | --- |
| | Patchy weak expression |
| | Patchy moderate expression |
| | Patchy strong expression |
| | Diffuse weak expression |
| | Diffuse moderate expression |
| | Diffuse strong expression |
| \*\*\* | Episomal HPV DNA |
| … | Integrated HPV DNA |

## Slide 2
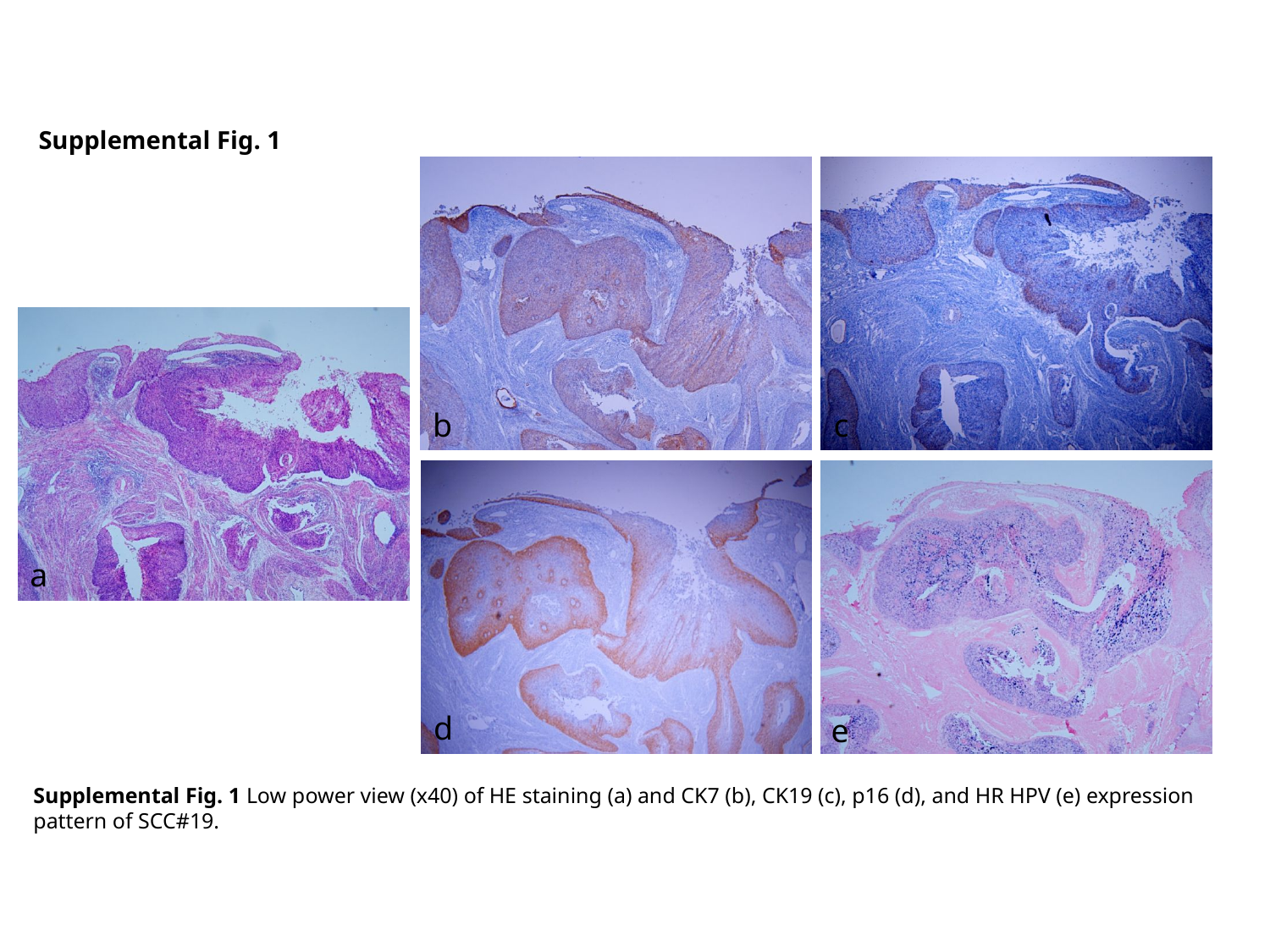

Supplemental Fig. 1
b
c
a
d
e
Supplemental Fig. 1 Low power view (x40) of HE staining (a) and CK7 (b), CK19 (c), p16 (d), and HR HPV (e) expression pattern of SCC#19.
