# Supplementary figures and images for: Erratum to: Cytokeratin7 and cytokeratin19 expression in high grade cervical intraepithelial neoplasm and squamous cell carcinoma and their possible association in cervical carcinogenesis
Source: Diagn Pathol. 2017 May 22;12:40. doi: 10.1186/s13000-017-0632-5 (PMC5440890; doi:10.1186/s13000-017-0632-5)

## Slide 1
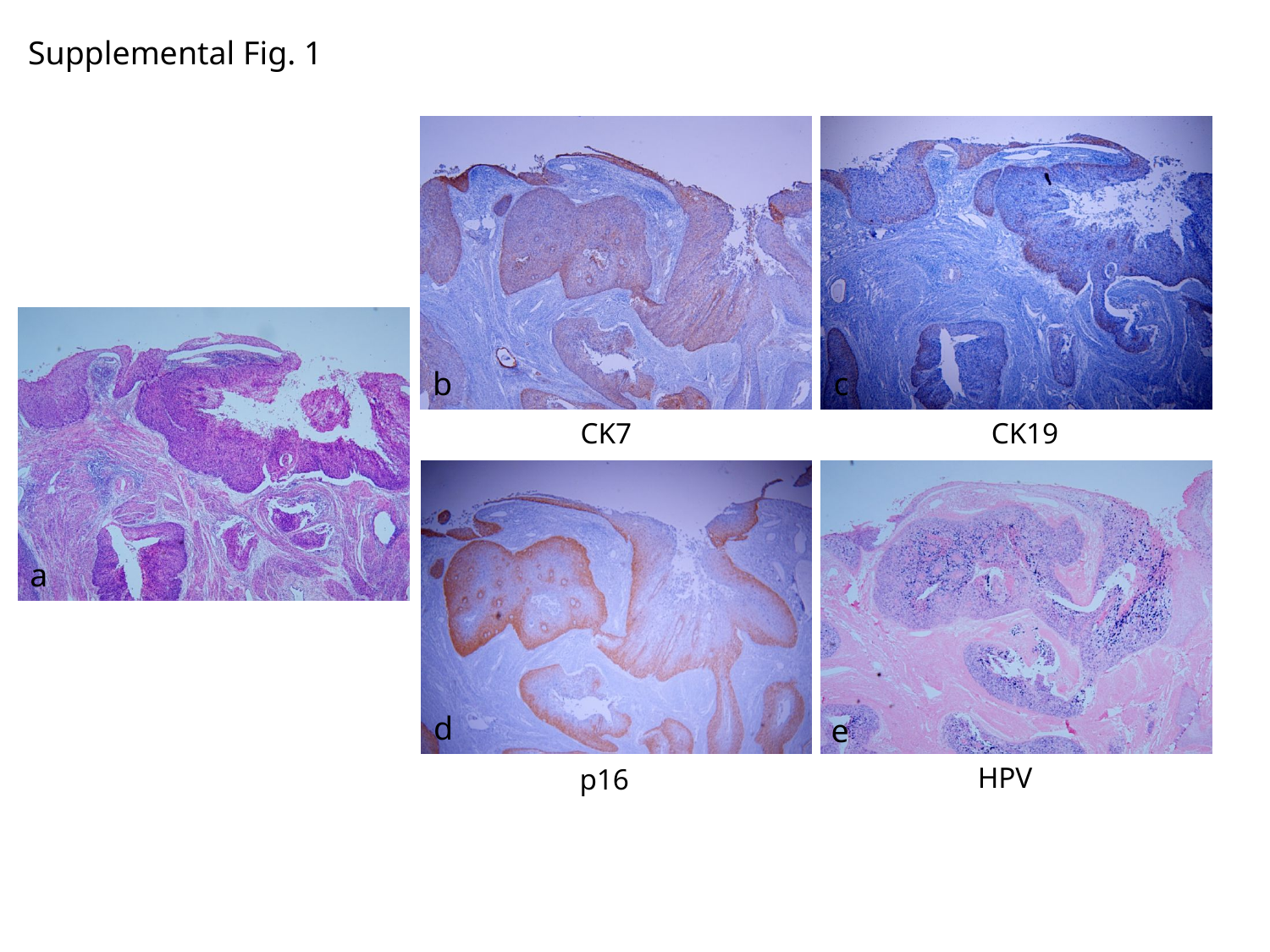

Supplemental Fig. 1
b
c
CK7
CK19
a
d
e
HPV
p16

Supplement: Additional file 1: Figure S1. — Low power view (x40) of HE staining (a) and CK7 (b) CK19 (c) p16 (d) and HR HPV (e) expression pattern of SCC#19 (PPTX 3720 kb) [file 13000_2017_632_MOESM1_ESM.pptx]
